# Functional Visual Symptoms, Accommodative Dysfunction, and Visual Performance Alterations in Chronic Work-Related Stress: A Narrative Review

**DOI:** 10.3390/vision10010014

**Published:** 2026-02-19

**Authors:** Mariaelena Malvasi, Elena Pacella, Simone De Sio, Gian Piero Covelli

**Affiliations:** 1 Department of Sense Organs, Sapienza University of Rome, 00185 Rome, Italy; elena.pacella@uniroma1.it (E.P.);; 2 Research Unit of Occupational Medicine, Sapienza University of Rome, 00185 Rome, Italy

**Keywords:** chronic stress, visual discomfort, asthenopia, visual function, autonomic nervous system, work-related stress, mobbing, visual performance

## Abstract

Background: Chronic work-related stress, including exposure to mobbing, is associated with a wide range of psychological and somatic consequences. However, its potential impact on visual function, particularly in the absence of structural ocular damage, remains underexplored. This narrative review critically examines the evidence linking chronic stress, autonomic nervous system (ANS) dysregulation, and functional visual disorders, focusing on accommodative function and asthenopia. Methods: A qualitative narrative review of the literature published between 2000 and 2025 was conducted using major biomedical databases. Studies addressing chronic stress, ANS activity, accommodative function, digital eye strain, and functional ocular symptoms were identified and integrated into a coherent pathophysiological framework. Results: The ocular system, being richly innervated by the ANS, may represent a peripheral target of prolonged stress-related autonomic alterations. Available evidence suggests that chronic stress is associated with asthenopia, accommodative inefficiency, and ocular discomfort even in the absence of overt ocular pathology. In particular, altered parasympathetic control of the ciliary muscle emerges as a plausible mediating mechanism. Conclusions: Functional visual disorders may represent peripheral manifestations of stress-related ANS dysregulation. Although causality cannot be established conclusively, the proposed framework supports the need for multidisciplinary research to clarify the clinical and medico-legal relevance of stress-related visual dysfunction.

## 1. Introduction

Chronic work-related stress represents a major public health issue, with well-documented repercussions on psychophysical health [1,2,3]. Among the most severe forms of occupational stress is mobbing, characterized by prolonged exposure to hostile, repetitive, and destabilizing behaviors [4,5], which may lead to anxiety–depressive disorders, autonomic dysregulation, and increased allostatic load [1,2,3].

In recent years, clinical attention has progressively expanded beyond the purely psychological consequences of stress to include the involvement of peripheral systems regulated by the autonomic nervous system. In this context, the visual system, and particularly the accommodative apparatus, may represent a functional target of chronic stress-related effects [6,7], although this relationship remains poorly explored within ophthalmological research.

Asthenopia and visual discomfort are frequently reported symptoms in individuals exposed to prolonged stress, even in the absence of structural ocular abnormalities [8]. The literature on digital eye strain and accommodative dysfunction suggests that these manifestations may arise from a dynamic dysregulation of focusing and convergence mechanisms, which are predominantly regulated by the autonomic nervous system. In particular, parasympathetic tone plays a central role in the control of the ciliary muscle and accommodative response, rendering this system especially sensitive to neurovegetative alterations associated with persistent stress [6,7].

### 1.1. Autonomic Regulation of Accommodation and Parasympathetic Tone

Accommodation is a dynamic visual function primarily regulated by the autonomic nervous system, with a predominant role played by parasympathetic innervation acting on the ciliary muscle through cholinergic pathways [6,7]. Parasympathetic activation induces ciliary muscle contraction, zonular fiber relaxation, and increased lens curvature, enabling near-vision focusing, whereas sympathetic activity plays a secondary modulatory role in accommodative relaxation and fine-tuning of the accommodative response [9,10].

Experimental and clinical studies have demonstrated that alterations in parasympathetic tone may significantly affect accommodative accuracy, stability, and flexibility [7]. Conditions characterized by autonomic imbalance, including chronic psychological stress, may lead to sustained parasympathetic activation or impaired sympathetic–parasympathetic interaction, resulting in accommodative spasm, increased accommodative lag, and reduced accommodative facility [11,12,13]. These mechanisms provide a plausible neurophysiological substrate for stress-related asthenopia in the absence of structural ocular abnormalities.

### 1.2. Measurement of Accommodation and Convergence in Clinical Practice

Accommodative and vergence functions can be assessed using both subjective and objective clinical methods. Commonly evaluated parameters include accommodative amplitude, accommodative lag, accommodative facility, and near point of convergence [14,15]. Objective techniques such as dynamic retinoscopy and open-field autorefractors allow assessment of accommodative response under natural viewing conditions, while subjective tests provide complementary information on functional performance and symptom correlation [15,16].

Reduced accommodative facility, increased accommodative lag, and convergence inefficiency have been consistently associated with visual fatigue and asthenopia, particularly during prolonged near tasks such as computer work, highlighting the importance of integrated binocular assessment in patients reporting functional visual discomfort [15,16,17,18].

### 1.3. Refractive Status, Age-Related Changes, and Visual Stress

Accommodation undergoes age-related changes, characterized by a progressive reduction in accommodative amplitude beginning around the age of 40 and culminating in presbyopia [19]. This physiological decline may interact with occupational visual demands, increasing susceptibility to visual fatigue and asthenopia in middle-aged and older individuals [19,20].

In addition, uncorrected or undercorrected refractive errors, particularly low hyperopia, astigmatism, and anisometropia, often impose an additional accommodative load, amplifying visual fatigue during prolonged near work [15]. These factors represent important confounding variables in the interpretation of stress-related visual symptoms and should be considered in both clinical practice and research settings [15,16].

### 1.4. Pharmacological Influences on Accommodation and Ocular Comfort

An additional variable influencing accommodative function and ocular comfort is the use of medications with anticholinergic properties, sympathomimetic agents, and several psychotropic drugs commonly prescribed [6,7]. Under stress-related conditions, these medications may alter autonomic balance, impair accommodation, reduce tear secretion, or increase ocular discomfort [21,22].

The functional effects of pharmacological agents on visual symptoms further underscore the multifactorial nature of functional visual disturbances and support the need for a comprehensive physiological framework when interpreting stress-associated accommodative alterations.

### 1.5. Pathophysiology of Asthenopia

Asthenopia primarily arises from functional overload of the accommodative and binocular systems rather than from structural ocular abnormalities [15,16,17,18]. Visual fatigue and discomfort associated with prolonged near work, including computer vision syndrome, have been consistently correlated with accommodative and vergence dysfunctions, such as reduced accommodative facility, increased accommodative lag, and convergence inefficiency [23,24,25].

Further clinical studies have shown that sustained visual demands may induce a state of persistent ciliary muscle activation, consistent with accommodative spasm, as well as a progressive reduction in the efficiency of the accommodative–vergence system [11,12,19]. These functional alterations are frequently observed in individuals reporting visual fatigue and are strongly correlated with subjective symptoms [18,25].

In parallel, psychosomatic and neurophysiological research has demonstrated that chronic stress may modulate the perception of ocular discomfort and pain through central and autonomic mechanisms, contributing to clinical entities described as functional ocular or visual pain [22,26,27]. However, an integrated interpretative framework systematically linking persistent occupational stress, autonomic regulation, and accommodative dysfunction remains incomplete.

While some functional visual symptoms may overlap with those reported after mild traumatic brain injury, any comparison should be strictly limited to functional and neurophysiological aspects, as stress-related asthenopia lacks evidence of structural brain damage [28].

Therefore, the aim of this narrative review is to critically analyze the available evidence on the relationship between chronic occupational stress, accommodative function, and functional visual symptoms, and to discuss the pathophysiological plausibility of interpreting these conditions as manifestations of neurofunctional dysregulation, without implying underlying anatomical damage, as well as their potential clinical and medico-legal implications.

### 1.6. Role of the Autonomic Nervous System in Visual Function

Mobbing in the workplace represents a complex condition characterized by hostile and repeated behaviors that generate profound psychological and social distress for affected individuals [4,5]. While the literature has extensively documented the psychological consequences, such as anxiety, depression, and stress-related disorders [1,2,3], as well as the psychosomatic impact of workplace harassment, the potential involvement of the visual system as a site of physical manifestation of damage remains largely unexplored and is entirely absent from the Italian regulatory framework.

From a legislative and medico-legal perspective, mobbing is generally associated with the recognition of psychological damage alone [4,5], whereas organic or functional damage to the visual system is not contemplated, despite increasing clinical observations suggesting a possible role of chronic stress in functional ocular disorders.

Recent evidence indicates that mental stress, anxiety, and prolonged distress may influence visual comfort, generate symptoms such as asthenopia and functional eye pain, and alter ocular function through neurovegetative and psychosomatic mechanisms [22,26,27,29].

### 1.7. Accommodative Implications of Prolonged Visual Stress

The central hypothesis of this review is that the accommodative system, predominantly regulated by the parasympathetic nervous system, may represent an effective “target organ” of chronic work-related stress. We propose that autonomic nervous system dysregulation induced by conditions such as bullying leads to persistent hyperactivation of the ciliary muscle, thereby compromising its functional efficiency and giving rise to asthenopic symptoms [6,7,11,12,29]. This pathophysiological sequence configures a potential functional ocular lesion that is not currently recognized from a medico-legal standpoint but is supported by growing neurophysiological evidence and warrants further clinical and regulatory investigation.

In this context, asthenopia and accommodative dysfunction may be conceptualized as core components of visual discomfort, reflecting the interaction between perceptual demands, neural regulation, and functional visual performance in everyday digital environments [23,24,25]. This hypothesis is intended as a conceptual framework rather than a causal model.

## 2. Materials and Methods

This study is based on a critical narrative review of the scientific literature aimed at analyzing the available evidence on the relationship between chronic stress, accommodative function, and functional visual symptoms. The purpose of this review was not to provide a quantitative estimate of causal associations, but rather to explore the physiological and clinical plausibility of the hypothesized mechanisms.

Relevant sources were identified through searches of major biomedical databases (PubMed/MEDLINE, MDPI, and Scopus), supplemented by selected open-access sources and grey literature. Articles published between 2000 and 2025 in English or Italian were considered eligible for inclusion.

The search strategy was based on combinations of keywords related to the main thematic areas of the review, including “chronic stress,” “work-related stress,” or “mobbing,” “autonomic nervous system,” “asthenopia,” and “visual function”. Boolean operators (AND/OR) were used to combine terms and broaden the retrieval of interdisciplinary literature.

We included review articles, observational studies, experimental studies, descriptive papers, neurophysiological contributions, and case reports relevant to the following domains: chronic stress and work-related stress; autonomic nervous system regulation and ocular function; accommodation, asthenopia, and digital eye strain; functional ocular disorders and non-organic ocular pain.

Exclusion criteria included articles not addressing visual or ocular outcomes, studies unrelated to occupational or chronic psychological stress, non-human studies (unless directly informative of autonomic or accommodative mechanisms), and publications lacking sufficient methodological or conceptual relevance to the scope of the review.

Within the narrative scope of this review, approximately 15–20 articles addressed the autonomic and neurophysiological control of accommodation, 20–25 studies examined associations between psychological stress and visual or ocular symptoms, 15–20 publications focused on accommodative dysfunction and asthenopia (including digital eye strain), and a smaller body of literature (approximately 8–12 studies) was considered for comparative models such as post-concussion syndrome and mild traumatic brain injury.

Selected contributions were analyzed using a qualitative and integrative approach, with particular attention paid to the proposed pathophysiological mechanisms, neurophysiological coherence, and clinical reproducibility of the reported observations. Percentages reported in the tables do not represent original data, but rather prevalence ranges derived from the literature and are used exclusively for descriptive and comparative purposes.

Given the narrative nature of the review and the lack of validated biomarkers for the objective assessment of stress-related visual dysfunctions, the findings should be interpreted as plausible pathophysiological hypotheses rather than as causal demonstrations. This review aims to provide a theoretical framework to support future prospective and multidisciplinary research.

## 3. Results

Based on the clinical observations reported in the literature and on the overall evidence analyzed, functional ocular manifestations appear to be particularly frequent [15,24,25]. In the context considered, the distribution of symptoms reflects a combined involvement of the ocular surface and the accommodative system. As shown in Table 1, conjunctival syndrome, tear film alterations, mild dry eye, and headache represent the most recurrent conditions, delineating a symptomatic profile consistent with that described in studies on digital eye strain and stress-induced visual disorders [15,24,25].

Prevalence ranges are derived from previously published studies on digital eye strain and stress-related visual symptoms [15,24,25].

Recent scientific literature has shown increasing interest in the relationship between psychological stress and ocular well-being. A 2024 study, Stress and Functional Eye Pain: How Mental Health Affects Eye Comfort, highlights how mental stress may generate sensations of pain, burning, fatigue, and ocular discomfort even in the absence of organic alterations [22]. This phenomenon is interpreted as the result of neuropathic mechanisms and dysfunction of peripheral neural pathways, suggesting that ocular symptom perception may be modulated by psychological and neurovegetative states [22,26,27].

As shown in Table 2, the most frequently reported ocular symptoms may be traced back to autonomic nervous system dysregulation and functional overload of the accommodative system, configuring a coherent pattern of stress-induced ocular vulnerability [12,15,18,22].

Isserow L.J. et al. [30] in their review, further highlight the association between chronic stress (acute, chronic, psychological, socioeconomic, and environmental) and the course of certain chronic ocular diseases, including glaucoma. According to the authors, prolonged stress may contribute to both the development and progression of ocular damage through the mechanism of allostatic load, defined as the physiological burden generated by continuous activation of stress-regulatory systems [31,32].

Similarly, Sabel et al. [33] in their review, further support the hypothesis that psychological factors such as stress, anxiety, and depression interact with neurophysiological and vascular processes involving the eye, contributing to visual deterioration. The authors propose a holistic clinical approach in which mental health and ocular health are jointly considered within a psychosomatic framework [29].

Another area of growing interest concerns digital eye strain. Numerous studies report that prolonged use of digital devices, combined with mental stress, reduced blink rate, sedentary lifestyle, and unfavorable environmental conditions, may significantly worsen ocular function and visual comfort [23,24,25,33]. These alterations not only affect visual well-being but may also have broader implications for mental health and overall quality of life [29,30,31,32,33].

Table 3 summarizes the main studies considered in the present review, highlighting study design, topics addressed, and key messages relevant to the interpretation of the relationship between chronic stress and functional visual disorders.

### Reported Symptoms and Hypothesized Mechanisms

The literature suggests the existence of multiple mechanisms through which chronic stress may interfere with visual function. Prolonged stress exposure has been shown to induce abnormal activation of the autonomic nervous system, with potential effects on ocular muscular and neural function [22,24]. This dysregulation may manifest as alterations in tear secretion, accommodation, corneal sensitivity, and overall visual perception, configuring a state of increased ocular vulnerability to adverse psychophysiological stimuli [29].

Another relevant pathophysiological element is the emergence of hypersensitivity within ocular neural pathways. This condition, described in studies on functional eye pain, is characterized by ocular pain or discomfort in the absence of evident organic lesions and reflects dysfunction of both peripheral and central sensory pathways [26,27]. This phenomenon appears particularly sensitive to psychological stress, which may amplify perceptual responses to minimal visual or somatic stimuli [22,26,27].

Moreover, several studies indicate that in the presence of predisposing conditions, such as uncorrected refractive errors, pre-existing ocular surface disorders, or intensive use of digital devices, stress may act as an aggravating factor [23,24,25]. The combination of ocular vulnerability and psychological stress tends to increase both the frequency and intensity of visual symptoms, leading to deterioration of visual comfort and perceived visual quality [33,34,35].

## 4. Discussion

This narrative review has critically examined the potential relationship between chronic work-related stress and functional visual disorders, with particular attention to accommodative dysfunction and asthenopia. Although visual symptoms have traditionally been considered marginal in stress-related disorders [15,29], the available evidence suggests that the visual system, and especially the accommodative apparatus, may represent a vulnerable functional target under conditions of persistent autonomic dysregulation [6,7,29].

Numerous studies indicate that prolonged exposure to psychological stress [15,20,21,29], such as that occurring in situations of workplace harassment, is associated with long-lasting alterations in autonomic nervous system balance [1,2,3,20,21,29]. These alterations are characterized by dysfunctional patterns of sympathetic and parasympathetic activity [6,7], leading to a state of functional parasympathetic predominance that disrupts the normal autonomic regulation of accommodation [9,10,29].

Given that the ciliary muscle is predominantly regulated by the parasympathetic nervous system, such dysregulation may plausibly interfere with the fine modulation of accommodation, resulting in reduced accommodative efficiency, increased accommodative lag, and decreased accommodative facility [5,11,12,16]. These alterations are consistent with the clinical manifestations of asthenopia and visual discomfort frequently reported by individuals exposed to chronic stress [15,16,17,18,22,23,24,25,29].

This mechanism, also described in psychosomatic stress conditions [22,29], suggests that asthenopia should not be regarded as a nonspecific symptom, but rather as a peripheral expression of systemic autonomic dysregulation [6,7,20,21]. From this perspective, impairment of ciliary muscle function represents the terminal phase of a stress-induced pathogenetic cascade grounded in well-established neurophysiological mechanisms [1,2,3,6,7].

It is noteworthy that these symptoms often occur in the absence of detectable structural ocular abnormalities [18,26,27]. This observation aligns with the concept of functional visual disorders, in which neurovegetative, perceptual, and central mechanisms contribute to symptom generation without demonstrable anatomical damage [26,27]. Within this framework, accommodative inefficiency may be interpreted as a peripheral manifestation of systemic autonomic dysregulation rather than as an isolated ocular pathology.

The hypothesis that asthenopia represents not a nonspecific complaint but the peripheral outcome of a stress-induced pathophysiological cascade is supported by evidence from both ophthalmological literature and psychosomatic research [22,29]. In particular, chronic stress–induced autonomic dysregulation may lead to persistent parasympathetic hyperactivity, resulting in sustained and non-physiological ciliary muscle contraction [11,12]. This condition compromises the dynamic ability to modulate focus, favoring the onset of fluctuating vision, headache, and asthenopia, in accordance with established pathophysiological models of accommodation [5,9,10,16].

In this context, the integration of evidence from studies on digital eye strain further strengthens the proposed interpretation [23,24,25,33,34,35]. These associations should be interpreted in light of relevant confounders, including screen time, age-related changes in accommodation, and individual susceptibility [19,20,23,24,25,40]. Prolonged engagement in near-vision tasks, especially under conditions of psychological stress, reduced blink rate, and tear film instability, may increase accommodative load and amplify ocular discomfort [23,24,25,33]. Stress may therefore act as an amplifying factor, increasing susceptibility to visual fatigue in individuals already exposed to high occupational visual demands [23,24,25,30,33,41,42].

Overall, the analyzed evidence suggests that the accommodative apparatus may plausibly be regarded, within a conceptual framework, as a potential peripheral target of prolonged occupational stress, with autonomic nervous system dysregulation acting as the main pathophysiological mediator. This interpretation is also consistent with the allostatic load model, according to which chronic exposure to stressors induces systemic functional alterations, involving peripheral districts that are particularly sensitive to autonomic modulation [1,3].

The reference to glaucoma in the table does not suggest a direct causal link with work-related stress. It is included to illustrate that stress-related autonomic dysregulation may influence ocular vulnerability and visual function in diseases known to be sensitive to systemic regulatory factors, without implying a causal relationship [31,32].

From a conceptual standpoint, the proposed model—chronic stress → autonomic nervous system dysregulation → accommodative dysfunction → asthenopia—may represent coherent and biologically plausible interpretative pathophysiological framework.

**Figure 1:**
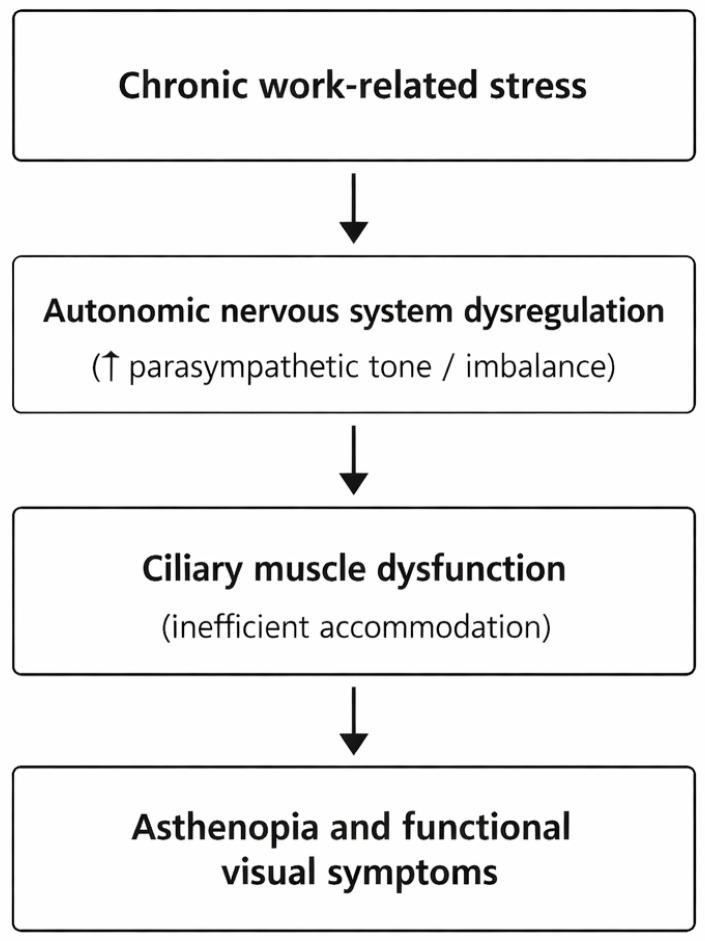
Conceptual model illustrating the proposed relationship between chronic work-related stress and functional visual symptoms. Prolonged psychological stress may induce autonomic nervous system dysregulation, characterized by altered sympathetic–parasympathetic balance and relative parasympathetic predominance (ANS). This imbalance may impair ciliary muscle function, leading to accommodative dysfunction and asthenopia, even in the absence of structural ocular damage.

The proposed interpretative pathway is schematically summarized in Figure 1.

However, it is essential to emphasize that the currently available evidence is predominantly associative in nature. The lack of validated biomarkers and standardized diagnostic criteria limits the possibility of rigorous objective assessment of stress-related visual dysfunctions.

Within this framework, visual discomfort emerges as a functional outcome of autonomic imbalance affecting accommodative control, rather than as a purely subjective complaint or a consequence of structural ocular damage.

From a broader medico-legal perspective, the issues discussed in this review are not unique to the Italian context but reflect more general challenges in the regulatory recognition of stress-related functional disorders. In many occupational health and compensation systems, medico-legal frameworks continue to prioritize demonstrable structural damage when defining ocular impairment [43], while functional and stress-related visual disturbances remain difficult to classify and formally recognize.

Within this context, the Italian regulatory framework is discussed as a representative example. At present, work-related stress conditions such as workplace bullying allow for the recognition of psychological injury, whereas organic or functional damage to the visual system is not explicitly contemplated. This discrepancy illustrates a broader gap between emerging clinical evidence on functional visual disorders and existing medico-legal standards, rather than a country-specific limitation.

Accordingly, the present review does not aim to redefine medico-legal criteria or compensation rules, but rather to highlight an evolving area in which clinical observations appear to precede regulatory adaptation. The increasing recognition of functional and autonomic-mediated disorders across medical disciplines suggests that stress-related visual symptoms may represent a relevant peripheral manifestation of chronic stress, warranting careful consideration in future clinical and medico-legal discussions.

These considerations underscore the need for continued interdisciplinary dialogue between clinical research, occupational health, and regulatory bodies, in order to bridge the gap between functional pathophysiological models and formal medico-legal frameworks.

Although patients with post-concussional syndrome and mild traumatic brain injury (TBI) may present visual symptoms that partially overlap with those observed in chronic work-related stress, such as photophobia, visual fatigue, and accommodative and vergence dysfunctions [28], in individuals with occupational stress these manifestations occur in the absence of any evidence of structural brain injury and should therefore be interpreted exclusively within a functional and neuroregulatory framework.

In this context, electrophysiological and functional assessment tools developed in traumatic brain injury research may offer useful methodological insights for future studies aimed at identifying objective markers of functional visual impairment, without implying nosological equivalence.

Looking forward, prospective and multidisciplinary studies integrating ophthalmological assessments, objective accommodative measurements, neurovegetative indicators, and psychological evaluation tools will be necessary. Such approaches may help clarify whether functional visual disorders represent a clinically relevant, reproducible, and potentially recognizable manifestation of chronic exposure to work-related stress.

Taken together, these observations support a speculative, hypothesis-generating model, rather than an emergent consensus, and should be interpreted accordingly

## 5. Conclusions

Based on the currently available evidence, chronic stress, including stress related to workplace bullying and harassment, may be associated with alterations in visual comfort and accommodative performance, contributing to asthenopic symptoms and other functional visual complaints. These manifestations are not currently recognized as structural ocular damage in medico-legal contexts and are more appropriately interpreted within a framework of systemic autonomic nervous system dysregulation, in which visual and accommodative disturbances represent functional expressions rather than organ-specific pathology [2,3,33].

The literature remains limited, particularly with regard to the lack of validated biomarkers, the difficulty in objectively quantifying functional visual alterations, and the small number of studies simultaneously assessing stress markers, autonomic indices, and accommodative function in populations exposed to chronic work-related stress.

At present, these limitations prevent the definition of a distinct visual disorder specifically induced by chronic work-related stress, as well as the establishment of a definitive clinical or medico-legal classification. Accordingly, prospective and multidisciplinary studies integrating ophthalmological evaluation, standardized accommodative and binocular measurements, autonomic indicators, and psychological assessment tools are needed. Within these boundaries, the proposed pathophysiological model should be regarded as an interpretative and hypothesis-generating framework, intended to guide future research aimed at improving symptom assessment and management, which are currently limited by the lack of validated scales and objective evaluation tools.

## Figures and Tables

**Table 1:** Prevalence of symptoms. Approximate prevalence ranges of visual and ocular symptoms reported in populations exposed to prolonged near-work or work-related stress. Values are derived from heterogeneous observational and clinical studies and are presented as indicative ranges rather than precise prevalence estimates. These data are intended to illustrate the variability reported in the literature and do not result from a systematic quantitative synthesis.

Symptom	Prevalence (%)
Conjunctival syndrome	40–45%
Chronic subclinical conjunctivitis	25%
Mild dry eye	20%
Headache	25%
Insomnia	20%
Tear film alteration	30–35%

**Table 2:** Integrated model for the interpretation of stress-related visual disorders (symptom → dysfunction → outcome): main clinical findings and hypothesized pathophysiological mechanisms. Reported values represent approximate ranges derived from heterogeneous sources and are provided for descriptive and illustrative purposes only, rather than as exact prevalence estimates or results of a systematic quantitative synthesis.

Clinical Finding	Pathophysiological Mechanism	Correlation with Stress	Implication
Lid lag	Reduced blink rate	Stress reduces blink frequency	Digital Eye Strain
Tear film alteration	Tear film instability	ANS alters secretion	Functional dry eye
Headache	Accommodative spasms	Parasympathetic hyperactivity	Asthenopia
Insomnia	Neurovegetative overload	Allostatic load	Increased ocular symptoms
Subclinical conjunctivitis	Functional micro-inflammation	Environment + stress	Digital Eye Strain/dry eye

**Table 3:** Summary of the Main Studies Considered in the Review.

Author/Year	Title	Study Type	Main Topics	Key Message
Isserow LJ et al., 2025 (MDPI)	Impact of Physiological and Psychological Stress on Glaucoma Development and Progression: A Narrative Review [30]	Narrative review	Allostatic load; glaucoma; chronic stress	Psychophysical stress contributes to the development and/or progression of glaucoma through neuroendocrine mechanisms.
Anderson, J. 2024 (Ophthalmology Case Reports)	Stress and Functional Eye Pain: How Mental Health Affects Eye Comfort [32]	Mini-review	Stress–glaucoma relationship; autonomic mechanisms; role of stress-reduction strategies	Psychological and physiological stress may influence glaucoma progression through mechanisms involving the autonomic nervous system, highlighting the relevance of stress in clinical management and disease understanding.
Sabel BA et al., 2018 (EPMA Journal)	Mental Stress as Consequence and Cause of Vision Loss [33]	Review	Mental stress ↔ vision loss; mind–body model	Visual deterioration has psychosomatic components, with stress acting both as a cause and a consequence of vision loss.
Kaur K et al., 2022 (Ophthalmology and Therapy)	Digital Eye Strain—A Comprehensive Review [34]	Clinical review	Digital eye strain; accommodation; visual fatigue	Dry eye syndrome is a multifactorial condition in which screen exposure, stress, and visual factors interact to exacerbate symptoms.
Coles-Brennan C et al., 2019 (Clin Exp Optom.)	Digital Eye Strain: A Comprehensive Review [35]	Clinical review	Dry eye syndrome; screen use; ergonomics	Prolonged digital device use leads to dry eye, blurred vision, and asthenopia, with stress acting as an aggravating factor.
MDPI, 2025 (Journal of Eye Movement Research)	A Review of Digital Eye Strain: Binocular Vision, Ocular Surface and Dry Eye Disease [36]	Review	Dry eye syndrome; pre-existing defects; ocular surface	Dry eye syndrome may exacerbate binocular vision anomalies, tear film instability, and accommodative dysfunctions.
MDPI, 2023 (Medicina)	Computer Vision Syndrome: An Ophthalmic Pathology of the Modern Era [37]	Review	Computer Vision Syndrome; multifactorial etiology	CVS is determined by visual, psychological, environmental, and behavioral factors.
MDPI, 2025 (Journal of Clinical Medicine)	Visual Functioning and Mental Health in the Digital Age [38]	Systematic review	Digital devices; mental health; visual symptoms	Asthenopia, visual fatigue, dry eye symptoms, blurred vision, headache, and ocular discomfort associated with prolonged digital device use and mental health factors.
Allied Academies, 2024	Understanding Functional Eye Pain [39]	Descriptive article	Functional ocular pain; neural hypersensitivity	Hypersensitivity of ocular neural pathways, modulated by stress, increases the perception of ocular pain.

## Data Availability

No new data were created or analyzed in this study.

## Abbreviations

The following abbreviations are used in this manuscript:

ANS	Autonomic nervous system
CVS	Computer Vision Syndrome
